# Aortic Thrombus Causing a Hypertensive Emergency

**DOI:** 10.5811/cpcem.2017.6.33876

**Published:** 2017-11-03

**Authors:** Kraftin E. Schreyer, Jenna Otter, Zachary Johnston

**Affiliations:** * Temple University Hospital, Department of Emergency Medicine, Philadelphia, Pennsylvania; † Lewis Katz School of Medicine at Temple University, Philadelphia, Pennsylvania

## Abstract

Thoracic aorta thrombi are a rare condition typically presenting as a source for distal embolization in elderly patients with atherosclerotic risk factors. However, young patients with a variety of presentations resulting from such thrombi have rarely been reported. We describe a case of a young patient with refractory hypertensive emergency caused by a large thoracic aorta thrombus. Investigation was guided by abnormal physical exam findings.

## INTRODUCTION

Thoracic aorta thrombi are exceedingly rare. When they do occur, they typically present as a source of distal embolization, clinically resulting in stroke, transient ischemic attack, or arterial embolization of an extremity. Diagnosis of thoracic aorta thrombi is often made in older patients with atherosclerotic risk factors or known aneurysmal or atherosclerotic disease.^1^,^2^ Thrombi have also been described in patients with known hypercoagulable states, infection, and trauma.^3^ We present a case of a 31-year-old human immunodeficiency virus (HIV) positive male with an aortic thrombus after remote endovascular treatment for a traumatic aortic injury.

## CASE REPORT

A 31-year-old Hispanic male with a history of HIV on highly active anti-retroviral therapy and prior endovascular stent placement in the thoracic aorta presented to the emergency department (ED) with leg weakness and shortness of breath. At the time of presentation, it was not known that the stent had been placed following a traumatic aortic dissection after a motor vehicle collision 13 years earlier. His other medical history included hypertension, small bowel obstruction, and a recent episode of *Pneumocystis jiroveci* pneumonia (PJP) complicated by ventilator-dependent respiratory failure.

On presentation, he complained of shortness of breath and leg weakness resulting in multiple falls over the preceding five days. His shortness of breath was continuous and aggravated by light activity including walking and supine positioning. It had progressively worsened over the course of one day and was associated with chest pain, subjective fever, and cough productive of blood-tinged sputum. According to the patient, he had experienced similar symptoms when he was diagnosed with PJP pneumonia.

On initial exam, the patient had a blood pressure (BP) of 247/128 mmHg, pulse of 135 beats per minute, and an oxygen saturation of 86% on room air. He was in distress, with labored breathing and decreased breath sounds bilaterally, with rhonchi auscultated in the lower lung fields. Although he was tachycardic, he was noted to be in a regular rhythm with normal heart sounds. He had palpable, strong radial pulses bilaterally. His abdomen was soft and non-tender without a pulsatile mass. He had full range of motion in his lower extremities with 5/5 strength and was ambulatory in the ED. He had no calf swelling, calf tenderness, or discoloration of the lower extremities. There was trace pedal edema bilaterally.

**Image 1:**
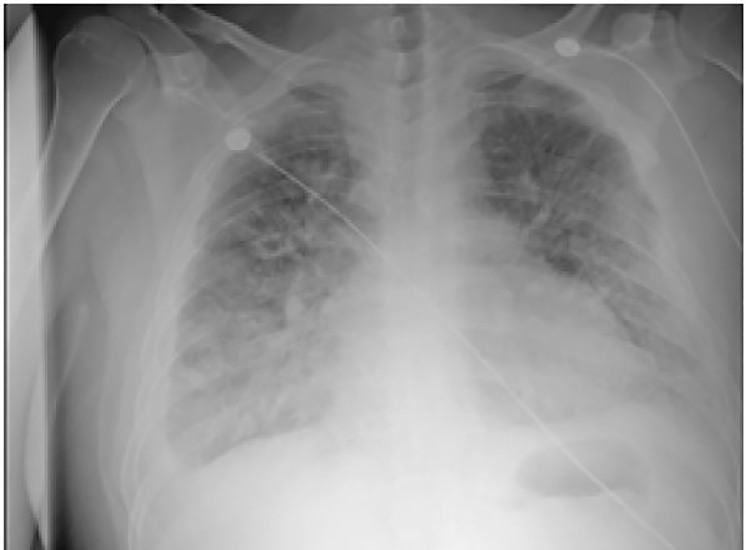
Initial anterior-posterior chest radiograph demonstrating diffusely hazy opacities in the lower lung zones and small bilateral pleural effusions, right larger than left.

An electrocardiogram revealed sinus tachycardia with lateral ST-segment depressions. Labs were notable for a sodium of 114mg/dL, a bicarbonate of 18mg/dL, and a creatinine of 1.3mg/dL. Troponin was elevated at 0.24ng/mL, brain natriuretic peptide level was 2346pg/mL, lactate dehydrogenase was 231U/L, and the results of an arterial blood gas demonstrated a pH of 7.41, a PaCO2 of 29mmHg, a PaO2 of 64mmHg, and a bicarbonate of 17.9mmol/L. All other labs were within normal limits. An initial chest radiograph showed diffusely hazy opacities in the lower lung zones (Image 1).

The patient was initially treated for sepsis from presumed PJP pneumonia and community-acquired pneumonia as well as hypertensive emergency. He was empirically treated with ceftriaxone, azithromycin, vancomycin, sulfamethoxazole/trimethoprim, and methylprednisolone. Attempts were made to lower his BP with intravenous labetalol with minimal improvement. His respiratory distress did not resolve and it was necessary to intubate the patient. He was admitted to the medical respiratory intensive care unit (ICU).

There, the patient continued to have refractory hypertension. Multiple agents were trialed, including maximal doses of nitroglycerin, nicardipine, metoprolol, clonidine and hydralazine, with no improvement. At that time, the patient’s mother arrived and provided additional history including the aforementioned stent placement.

**Image 2:**
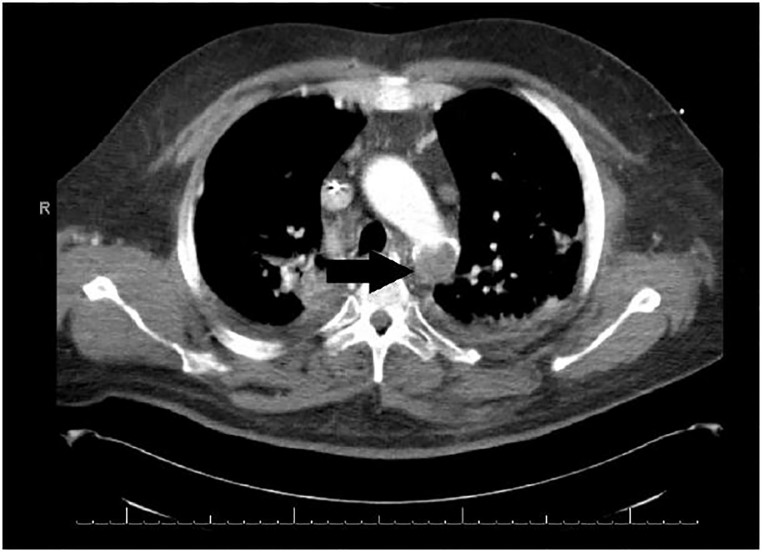
Computed tomographic angiography in axial view demonstrates complete occlusion of the proximal descending thoracic aortic stent (arrow).

**Image 3:**
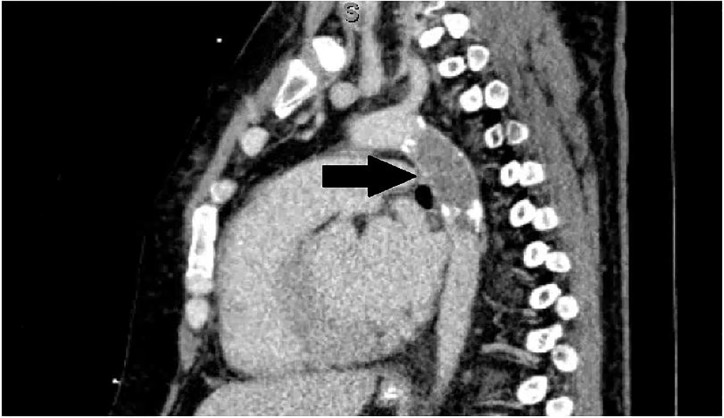
Computed tomographic angiography of the chest in sagittal view demonstrates complete occlusion of the proximal descending thoracic aortic stent (arrow).

In light of the additional history, four extremity BPs were obtained to evaluate for possible vascular injury or compromise. BPs were found to be 195/70mmHg in the bilateral upper extremities and 70/40mmHg in the bilateral lower extremities. Upper and lower extremity arterial lines were placed and confirmed the BP discrepancies. A computed tomography angiography (CTA) study revealed a complete occlusion of the thoracic aorta at the site of prior endovascular stent placement (Image 2 and Image 3).

The patient was transferred to the cardiac ICU under the care of thoracic surgery where he underwent operative repair with axillary-femoral arterial bypass. Unfortunately, the thrombus had led to multisystem organ failure with cerebral edema, pulmonary edema, congestive heart failure, ischemic hepatitis, ischemic bowel, renal failure requiring continuous veno-venous hemodialysis, and severe rhabdomyolysis. He was transferred to the surgical ICU where he died from complications related to multiple organ failure.

**CPC-EM Capsule**

What do we already know about this clinical entity?

Thoracic aorta thrombi are exceedingly rare. When they do occur, they typically present as a source of distal embolization in a patient with known atherosclerotic disease.

What makes this presentation of disease reportable?

This case highlights an unusual presentation of an aorta thrombus in a young patient, causing hypertensive emergency leading to multi-organ failure.

What is the major learning point?

This case reminds the clinician to keep a broad differential diagnosis, do a thorough history and physical exam, and consider cognitive biases in every case.

How might this improve emergency medicine practice?

Recognizing the constellation of risk factors and history and physical exam findings can lead to an early diagnosis and expedite life-saving interventions.

## DISCUSSION

Thoracic aorta thrombi are extremely rare. There are only approximately 100 cases reported, most of which involve a mobile thrombus in the aortic arch resulting in embolization.^1^,^4^,^5^,^6^ Fifty percent of patients present with stroke, 35% with transient ischemic attack, and 14% with signs and symptoms of peripheral emboli.^1^,^5^ Gagliardi et al. reviewed an additional 14 cases and demonstrated a link between aortic thrombi and acute lower extremity ischemia in 12 of those cases, and a link to chronic lower extremity ischemia in the other two.^6^ Our case involves a patient presenting with what initially appeared to be PJP causing respiratory failure, later recognized as an acute on chronic thoracic mural thrombus causing multisystem organ failure. Our patient was at high risk for a thrombus based on his prior surgery, which resulted in endothelial damage to the aorta. Along with inflammation and alteration in flow, this created Virchow’s triad of hypercoagulability.

He was effectively suffering from a complete coarctation of the aorta, which led to hyperperfusion proximal and hypoperfusion distal to the thrombus. Aortic thrombi are rare, and the authors are aware of only one other case similar to that presented here. Lin et al.^7^ described a case of aortic sarcoma presenting in a comparable fashion to that of our patient.

The thrombus occluded his thoracic aorta and resulted in higher pressures proximal to the obstruction. This was likely compounded by the activation of the renin-angiotensin system, triggered by hypoperfusion of the kidneys. Elevated pressures proximal to the thrombus likely led to elevated cerebral perfusion pressures and pulmonary hypertension, leading to cerebral and pulmonary edema. It also caused the markedly elevated BPs in the upper extremities, which were what led to his initial diagnosis of hypertensive crisis.

Organs below the obstructed aortic lumen suffered from hypoperfusion, which led to ischemic hepatitis, renal failure, ischemic bowel, peripheral arterial ischemia and subsequent rhabdomyolysis. The patient’s lower extremity pulses were diminished, and following CTA it was determined that nearly all of his vascular supply distal to the occlusion was via collateral vessels. We additionally believe that the low-flow state of his lower extremities led him to experience subjective weakness, which he complained of on initial presentation.

Many factors were at work against the clinician in the ED when this patient first presented. He initially presented in extremis, requiring emergent airway stabilization with intubation. This precluded the clinician from obtaining a full history from the patient, as he came to the ED unaccompanied. Moreover, the patient attributed his symptoms to another pathology. These circumstances likely led the clinician to fall subject to unpacking bias, in which a complete history was not obtained, and to anchoring bias, in which one becomes attached to a particular diagnosis, such as the one proposed by the patient, early on in the clinical course. Had the clinician avoided these biases, he or she might have considered a vascular etiology and assessed four extremity blood pressures and pulses. This crucial exam finding was key in eventually determining the diagnosis via CTA of his chest. In this case, cognitive biases, diagnostic momentum and consequent diagnostic delay contributed to his death.

## CONCLUSION

We present a rare case of intramural aortic thrombus, masquerading as PJP pneumonia in a young HIV-positive patient. This rare case should remind the clinician in the ED to consider the words of the patient, but not to anchor on the patient’s belief as to the etiology of his or her complaint. Additionally, it highlights the importance of a full history and physical exam, and a broad differential diagnosis. In this case, cognitive biases, including unpacking, anchoring, and diagnostic momentum caused a delay in definitive surgical management of this patient’s aortic pathology. Late additions to the patient’s medical history, red flags in the patient’s physical exam, and symptoms refractory to treatment eventually led to reevaluation with four extremity pulses and blood pressures. But, by that time, the self-perpetuating cycle of hyperperfusion proximal and hypoperfusion distal to the thrombus had irreparably damaged his organs.

In the ED we are focused on the management mantra of the ABC-driven (airway, breathing, circulation) assessment of a critical patient, and are too familiar with the adage “when you hear hoofbeats, think horses, not zebras.” This zebra reminds us to keep a broad differential diagnosis and reevaluate a patient if aspects of a case arise that do not fit the working diagnosis.
